# Antioxidant Effects of Irisin in Liver Diseases: Mechanistic Insights

**DOI:** 10.1155/2022/3563518

**Published:** 2022-01-07

**Authors:** Junzhou Zhao, Linlan Qiao, Jian Dong, Rongqian Wu

**Affiliations:** ^1^National Local Joint Engineering Research Center for Precision Surgery & Regenerative Medicine, Shaanxi Provincial Center for Regenerative Medicine and Surgical Engineering, First Affiliated Hospital of Xi'an Jiaotong University, Xi'an, Shaanxi Province, China; ^2^Department of Hepatobiliary Surgery, First Affiliated Hospital of Xi'an Jiaotong University, Xi'an, Shaanxi Province, China

## Abstract

Oxidative stress is a crucial factor in the development of various liver diseases. Irisin, a metabolic hormone discovered in 2012, is mainly produced by proteolytic cleavage of fibronectin type III domain containing 5 (FNDC5) in skeletal muscles. Irisin is induced by physical exercise, and a rapidly growing body of literature suggests that irisin is, at least partially, responsible for the beneficial effects of regular exercise. The major biological function of irisin is believed to be involved in the maintenance of metabolic homeostasis. However, recent studies have suggested the therapeutic potential of irisin against a variety of liver diseases involving its antioxidative function. In this review, we aim to summarize the accumulating evidence demonstrating the antioxidative effects of irisin in liver diseases, with an emphasis on the current understanding of the potential molecular mechanisms.

## 1. Introduction

**Figure 1:**
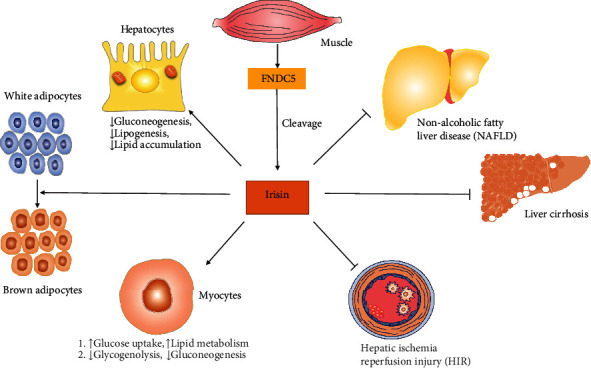
Pleiotropic traits of irisin.

Irisin is a metabolic hormone discovered by Spiegelman's group in 2012 [1]. It consists of 112 amino acid residues and is mainly produced by cleavage of fibronectin type III domain containing 5 (FNDC5) in response to exercise. A rapidly growing body of literature suggests that irisin is at least partially responsible for the beneficial effects of regular exercise, such as weight loss, stronger bones, and better cardiovascular health [2, 3]. The major biological function of irisin is believed to be involved in the maintenance of metabolic homeostasis. In adipose tissues, irisin induces browning of white adipose tissue to increase energy expenditure [4]. In myocytes, irisin improves fatty acid oxidation and glucose utilization [5]. In hepatocytes, irisin suppresses gluconeogenesis, lipogenesis, and lipid accumulation [6, 7]. However, recent studies have suggested that irisin has potent antioxidative properties and is expected to be a potential therapeutic agent to protect tissues from oxidative stress-induced injury [8]. Oxidative stress occurs when the production of free radicals overwhelms the antioxidant system. The imbalance between oxidants and antioxidants can lead to structural and functional damage in the liver [9]. Increased oxidative stress is the underlying mechanism of many liver diseases, regardless of the cause of the liver disorder [10]. Oxidative stress is a crucial factor in the development of various liver diseases, including nonalcoholic fatty liver disease (NAFLD), hepatic ischemia reperfusion injury (HIR), and liver cirrhosis (Figure 1). Considering the global burden of liver diseases, herein, we mainly discuss the antioxidative effects of irisin in the liver. We aim to summarize the accumulating evidence demonstrating the antioxidative effects of irisin in liver diseases, with an emphasis on the current understanding of the potential molecular mechanisms (Table 1). In this paper, we will firstly discuss mechanisms of irisin's antioxidant action, then signaling pathways related to the antioxidant effect of irisin in the liver, and finally the conclusion of this review.

## 2. Mechanisms of Irisin's Antioxidant Action

The reduction in ROS production is due to the antioxidant effect of irisin. Understanding the approach of ROS production is helpful to comprehend the antioxidant mechanism of irisin. Free radicals are unstable and highly reactive atoms or molecules with unpaired electrons. There are two types of free radicals, reactive oxygen species (ROS) and reactive nitrogen species (RNS). ROS can be generated by intracellular and extracellular sources. Mitochondria are a major intracellular source for the production of ROS. In mitochondria, during the processes of electron transport to O2, variable degrees of reactive intermediates are formed, including ROS. Some redox-related enzymes (NO synthetase, xanthine oxidase, etc.) also produce ROS. In addition, macrophages and other monocytes contribute to the manufacture of ROS. The extracellular sources of ROS are mainly radiation, chemicals, and some virus infection [11]. Indeed, ROS are involved in a series of physiological processes, including signal transduction, microorganism eradication, and programmed cell death, all of which are essential for homeostasis stabilization. However, in most situations, high levels of ROS can oxidize all molecules in the cell membrane and contents, causing severe gene damage, and eventually triggering cell death by an apoptotic mechanism [12]. Unfortunately, the liver is the main attack target of free radicals. Intriguingly, we observed that, in hepatic ischemia reperfusion injury, hepatocytes express less antioxidant enzymes (superoxide dismutase (SOD) and glutathione (GSH)) [13, 14].

The antioxidant mechanism of irisin, in a broad sense, means not only the reduction of ROS but the decrease the consequential complications. The current literature showed that the antioxidant effect of irisin is associated with regulating several vital cellular processes, including modulating mitochondrial fission and fusion, inhibiting inflammasome activation, improving autophagy, suppressing ER stress and ferroptosis, and reversing cell death. Among the above cellular processes, modulating mitochondrial fission and fusion as well as improving autophagy is involved in reducing ROS production, whereas the processes such as inflammasome activation, ER stress, ferroptosis, and cell death are all the consequential complications of ROS damage. These ROS damage complications would paradoxically enhance the ROS production provided they are not eliminated [15]. In other words, controlling complications of ROS damage plays a dominating role in addressing oxidative stress.

### 2.1. Irisin Improves Mitochondrial Dynamics

Mitochondrial fission and fusion participate in regulating mitochondrial function and are controlled by mitochondrial fusion and fission-related genes, including Mitofusin1, 2 (Mfn1, 2), Dynamin-related protein 1 (Drp1), and Mitochondrial fission 1 protein (Fis1) [14]. Mitochondrial biogenesis (TFAM and PGC-1a gene controlled) is associated with selective inheritance, and functional mitochondrial biogenesis would limit the transmission of damaged mitochondrial genetic materials [16]. The balance between mitochondrial fission and fusion plays an important role in the maintenance of normal mitochondrial function. Previous research has shown that abnormal mitochondrial fission or fusion was a predictor of some diseases that are associated with oxidative stress and mitochondrial damage. Wu et al. suggested that quantum dots (QDs) might cause mitochondrial damage and overproduction of ROS by inducing imbalanced mitochondrial fission/fusion in HepG2 cells [17]. On the contrary, another study found that the upregulation of mitochondrial fusion led to less ROS production, which further validated the importance of mitochondrial fission/fusion balance [18]. However, enhanced mitochondrial biogenesis promotes hepatocyte epithelial mesenchymal transition [19]. What is more, the restoration of mitochondrial dynamics participated in the suppression of oxidative stress. Das et al. demonstrated that melatonin helped to decrease ROS production by reducing abnormal mitochondrial dynamics [20]. Irisin modulates mitochondrial fusion, fission, and biogenesis to suppress oxidative stress. We found that irisin improved mitochondrial condition, exhibiting increased numbers of mitochondria and reduced mitochondrial fission or fusion. And irisin increased mitochondrial biogenesis [14]. Furthermore, we observed irisin significantly decreased ROS levels and decrease the inflammatory cytokines (ALT, AST, LDH) after liver ischemia-reperfusion injury, which may be strongly associated with mitochondrial function improvement [14].

### 2.2. Irisin Downregulates Inflammasome

The inflammasome is recognized as one of the culprits of the oxidative stress damage [21]. In general, inflammasomes can mediate host immune responses to bacterial and cellular damage as forms of cytoplasmic protein complexes [21]. However, in pathological condition, inflammasome activation is a severe complication of oxidative stress and could cause cell death and tissue damage. Especially, the NLRP3 inflammasome can trigger the activation of caspase-1 to convert the immature cytokines pro-IL-1b and pro-IL-18 into mature cytokines IL-1b and IL18, respectively, to cause inflammatory damage. In addition, inflammasome complex activation is associated with liver inflammation and fibrosis. Shi et al. found that, in diabetic C57BL/6 mice, ROS-mediated NLRP3 inflammasome activation induced massive hepatocyte pyroptosis [22]. And it is also reported that carnitine palmitoyltransferase 1a (Cpt1a) would exacerbate ROS-induced inflammatory damage via the inflammasome activation signaling pathway in liver injury, which further demonstrated the tight relation between inflammasome activation and ROS damage [23]. The downregulation of inflammasome activation can mitigate ROS-induced inflammatory damage, exemplifying by a study in which allicin could reduce inflammasome activation to alleviate liver injury by suppressing hepatic oxidative stress [24]. Interestingly, another research discovered that NLRP3 inflammasome activation was downregulated when the ROS level was reduced by ginsenoside Rg1 in alcoholic hepatitis, implying the interaction between inflammasome activation and oxidative stress [25]. Moreover, Dong et al. indicated that the inhibition of inflammasome activation relied on the ROS-related pathway in nonalcoholic steatohepatitis [26]. Inflammasome activation is a major target of irisin to ameliorate oxidative damage in liver disease. Irisin inhibits inflammasome activation to protect against LPS-induced liver injury by reducing the ROS signaling pathway [15]. Fan et al. suggested that irisin participated in the hepatoprotection of dexmedetomidine by both suppressing oxidative stress and inhibiting inflammasome activation [27]. The irisin antibody partly abrogated the hepatoprotection of dexmedetomidine, which further confirmed the function of irisin [27]. Although the definite mechanism is unknown, it is hypothesized that dexmedetomidine promotes irisin to be grabbed from other body tissues, to be poured into blood circulation, and then to be transferred into liver tissue by dexmedetomidine [27]. Therefore, irisin level would be elevated in both serum and liver. Some studies found that, in cerebral ischemia mice, the low irisin level may be as a result of low-level physical exercise (brain damage-induced) [27, 28].

### 2.3. Irisin Decreases ER Stress

The endoplasmic reticulum (ER) is an essential organelle, possessing the majority of calcium and controlling protein translation [29]. The ER will respond to any disturbance of its normal function as well as the related signaling pathway, which is called ER stress [29]. And ER stress is associated with ROS level. A research found that both ER stress and ROS levels were increased in visfatin-treated livers and accompanied by hepatic damage [30]. Sharma et al. found that in hepatic stellate cells, ER stress and oxidative stress were induced by sorafenib to cause hepatic cell death, giving an evidence that ER stress was consistent with oxidative stress to cause liver damage together [31]. Previous research showed that decreasing ER stress levels was related to the inhibition of ROS production. An et al. indicated that black ginseng reduced ROS production in NAFLD through an ER stress-related pathway [32]. And another research observed that rosmarinic acid alleviated acrylamide-induced hepatic oxidative stress and decreased ER stress, which also implied that ER stress was a potential modulator of ROS production [33]. Irisin can regulate ER stress to attenuate hepatic oxidative stress. We found that irisin could significantly alleviate oxidative stress and ER stress in mice with acute pancreatitis [34]. Irisin may attenuate pancreatic fibrosis by reducing oxidative and ER stress [35]. In mice with liver steatosis, irisin also improves liver function and decreases ER stress and ROS production [36]. The irisin exerts a consistent function in oxidative stress and ER stress, indicating that ER stress is, at least in part, associated with antioxidant mechanism of irisin.

### 2.4. Irisin Promotes Autophagy

Autophagy plays a crucial role in a wide variety of pathological processes, including oxidative stress [37]. The overproduction of ROS will cause mitochondrial dysfunction and lipid peroxidation to break cellular homeostasis, in which autophagy may be induced [37]. It is found that autophagy participated in the repair of ROS-induced oxidative proteins in LO2 cells [38]. Chang et al. found that the production of ROS was elevated in diTFPP/C2-ceramide-treated cells with the activation of autophagy, proving autophagy is involved in the oxidative processes [39]. The induction of autophagy also helps to suppress oxidative stress. A recent scientific study suggested that dihydrokaempferol reduced the production of ROS to attenuate liver injury by promoting autophagy [40]. Moreover, it is reported that 5-O-demethylnobiletin enhanced autophagy to mitigate oxidative stress in CCl_4_-induced acute liver injury, which further evidences that autophagy could ameliorate the ROS production [41]. Autophagy is a target of irisin to exert antioxidant effects. Specifically, irisin enhances the activation of autophagy to reduce mitochondrial permeability transition and then to decrease ROS levels. Li et al. used INS-1 cells as experimental models, revealing that metformin could elevate irisin expression and autophagy function to suppress cell apoptosis [42]. We used male Sprague-Dawley rats as animal models, observing that irisin would increase telomerase activity, autophagy, and improve oxidative stress in rats HIR. However, the inhibition of autophagy attenuated the irisin's hepatoprotective/antioxidant effects after rats HIR, further verifying the autophagy's vital role in antioxidant effect of irisin [13].

### 2.5. Irisin Decreases Cell Death

Cell death (apoptosis/ferropotosis) is the definitive result of oxidative stress. In the early stage of apoptosis, the cellular ROS level is increased [43]. The overproduction of ROS will cause cellular redox status imbalance and organelle damage, leading to cell death eventually [43]. And it is discovered that Moringa oleifera fruit could cause ROS-mediated apoptosis in human liver cancer HepG2 cells [44]. What is more, Wang et al. demonstrated that EPS364, a deep-sea bacterial exopolysaccharide, exhibited an antitumor trait by inducing hepatic cell death by upregulating ROS production, supporting the idea that oxidative stress could trigger cell death [45]. And the idea is likewise proofed by another research where diTFPP/C2-ceramide increased hepatic cell death by increasing ROS levels [39]. Evidence has shown that the inhibition of ROS production can alleviate abnormal cell death. Hussein and colleagues found that chlorogenic acid, quercetin, coenzyme Q10, and silymarin reduced hepatic cell death and decreased ROS production [46]. Furthermore, another scientific research reported that Kushenol C reduced cell death by downregulating ROS levels and upregulating antioxidant enzyme expression, giving a more direct evidence that less oxidative stress induced less cell death [47]. Irisin exhibited antioxidant effects by improving abnormal cell death. Li et al. demonstrated that irisin suppressed oxidative stress and decreased cell death in LPS-induced liver injury [15]. It is also reported that the upregulation of hepatic irisin negatively regulated cell death and oxidative stress [48]. We also found that irisin improved autophagy to attenuate liver damage by decreasing ROS production and related cell death [13].

## 3. Molecular Basis of Irisin's Antioxidant Effect

**Figure 2:**
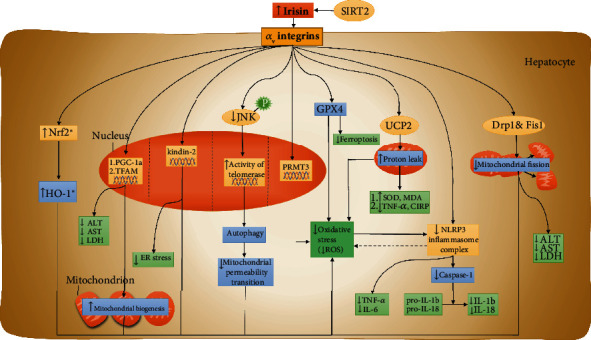
Signaling pathways of antioxidant effects of irisin in hepatocytes.

According to the current literature, irisin might alleviate the oxidative stress through above cellular processes modulation by regulating several intricate signaling pathways. And the core molecules in these signaling pathway are *α*_v_ integrins, UCP-2, PRMT3, telomerase, Nrf2, Kindlin-2, JNK, GPX4, and SIRT2. Interpretation of the molecular basis of these signaling pathway is helpful to profoundly understand the mechanism of the antioxidant effect of irisin (Figure 2).

### 3.1. *α*_v_ Integrin Is the Transmembrane Receptor of Irisin

Integrins, a family of transmembrane cell adhesion molecules, take part in mediating cell-matrix and intercellular interactions [49]. *α*_v_ integrins are pivots that connect fibroblasts, extracellular matrix, and monocytes [49]. Furthermore, in liver disease, *α*_v_ integrins are crucial molecules in the development of liver fibrosis. And it is also reported that *α*_v_ integrins regulated procollagen production in human hepatic stellate cells [50]. And a recent study likewise indicated that in *α*_v_ integrin-knockout mice, the development of fibrosis was dampened, giving a further verification that *α*_v_ integrins participated in the development of liver disease [51]. The inhibition of the expression of *α*_v_ integrins will improve liver fibrosis. Zhang et al. suggested that the biphenyl compound IMB-S7 ameliorated liver fibrosis by downregulating *α*_v_ integrin levels [52]. The *α*_v_ integrins are also involved in the metastasis of cancer. Previous research demonstrated that fucoidan-Sargassum inhibited liver cancer metastasis by activating the *α*V*β*3/Src/E2F1 signaling pathway [53]. Oxidative stress induces high levels of inflammatory cytokines, which affect the expression of *α*_v_ integrins. It is reported that IL-8 promoted the expression of *α*_v_ integrins through the PI3K/Akt pathway in hepatocellular carcinoma [54]. The *α*_v_ integrins, as an irisin transmembrane receptor, served the vital role in the antioxidant effect of irisin in liver disease. We found that irisin binds with *α*_v_ integrins to suppress oxidative stress in mice with HIR injury [36, 55] (Figure 2). Although *α*_v_ integrins are involved in the development of liver disease and participate in the antioxidant effect of irisin, there are still no direct evidences showing that the regulation of the *α*_v_ integrins could affect the ROS production by irisin [36]. Many important questions about *α*_v_ integrins, irisin, and their interaction remain unanswered and need further research.

### 3.2. Irisin Upregulates UCP-2

Mitochondrial proton leakage has a major impact on controlling ROS production [56]. Proton leakage allows a small amount of proton to flow back to the matrix to decrease the chemical gradients and electrical gradients [56]. Uncoupling protein-2 (UCP-2) belongs to the family of mitochondrial anion carrier proteins and is expressed in various tissues, including the liver [57]. UCP-2 can decrease ROS emission from mitochondria by catalyzing proton leakage [58]. The uncoupling function and ROS-eliminating mechanism of UCP-2 have been well studied. It is found that inhibition of UCP-2 causes a rapid increase in H_2_O_2_ production. In UCP-2 knockout mice, Arsenijevic et al. showed that the phagocytosis function of macrophages is enhanced due to the increased production of ROS in mitochondria [59]. Several studies have demonstrated the critical role of UCP-2 in irisin activity. Chen et al. showed that irisin stabilizes UCP-2 in the mitochondria and reduces its degradation, which in turn results in preservation of mitochondrial metabolism and protection from I/R-induced oxidative stress. We also found that irisin suppressed oxidative stress and upregulates UCP-2 expression after hepatic I/R in C57BL/6J mice [14] (Figure 2). In addition, we observed that irisin decreased the inflammatory cytokines (TNF-*α*, CIRP) and increased the antioxidant enzymes level (SOD, MDA) [14]. The antioxidative effect of irisin was significantly attenuated after the administration of genipin (an inhibitor of UCP2), which demonstrates that UCP-2 is essential for the antioxidative activity of irisin [14].

### 3.3. Irisin Attenuates the Function of PRMT3

We found that liver oxidative injury was worse in HFD-induced, high serum fatty acid mice than in control group mice, suggesting that lipid contents fueled oxidative stress and that the inhibition of lipogenesis may play an important role in the antioxidant effect of irisin [14]. A recent study by Hoekstra and colleagues showed that the inhibition of protein arginine methyltransferases (PRMTs) holds down hepatic lipogenesis [60]. Moreover, previous research found that PRMT3 activation could be an inducer of fatty liver by binding to liver X receptor *α* (LXR*α*), which further supported that PRMT3 participated in lipogenesis [61]. Park and colleagues found indirect evidence that PRMT3 participates in the antioxidant effect of irisin [62]. They observed that in palmitic acid- (PA-) treated mice, ROS levels, and lipogenic mediators were high [62]. High ROS levels are always followed by excessive inflammatory cytokine levels. Intriguingly, irisin reduced the expression of PRMT3 and ROS production as well as lipogenesis in PA-induced hepatic steatosis; however, the overexpression of PRMT3 reversed the antilipogenesis and anti-inflammatory function of irisin [62] (Figure 2). Although there was no direct evidence indicating that irisin alleviated oxidative stress by downregulating PRMT3 expression, at least in part, PRMT3 was involved in the antioxidant effect of irisin because PRMT3 played an important role in suppressing inflammatory stress, the chain reaction of oxidative stress [62].

### 3.4. Irisin Elevates the Function of Telomerase

Telomerase is a key enzyme responsible for maintaining the length of telomeres [63]. The activity of telomerase is associated with carcinogenesis and oxidative stress. Decreasing telomerase activity is present in liver fibrosis and hepatocyte senescence; however, increasing telomerase activity is seen in liver cancer [64]. In cancer cells, upregulation of ROS suppresses telomerase activity. Antioxidants protected telomerase activity in normal cells but paradoxically inhibited telomerase activity in cancer cells; however, the underlying mechanism is still unknown. Similarly, a recent literature observed that NAC, an antioxidant, decreased ROS levels and decreased telomerase activity, inducing cancer cell death [65]. Telomerase also participates in the regulation of autophagy. It is reported that downregulation of telomerase activity impaired autophagy function, leading to the overproduction of ROS [66]. Moreover, Green et al. also demonstrated that telomerase participated in the cellular response to oxidative stress by regulating autophagy, giving a further verification about interaction between telomerase and autophagy [67]. Irisin suppresses oxidative stress in a telomerase/autophagy-dependent manner. We found that irisin decreased the production of ROS by restoring autophagy function by upregulating telomerase activity [13]. After the administration of the telomerase inhibitor BIBP1532, the antioxidative effect of irisin was dampened, further confirming telomerase function [13] (Figure 2).

### 3.5. Irisin Promotes Nrf2 Signaling

Nuclear factor-E2-related factor 2 (Nrf2) is a crucial regulator of cellular redox homeostasis [11]. The inhibition of Nrf2 signaling is associated with increased ROS production [11]. Luo et al. demonstrated that the overexpression of carnitine palmitoyltransferase 1 stimulated the production of ROS in liver injury by suppressing the Nrf2/HO-1 signaling pathway [23]. Conversely, the activation of Nrf2 can ameliorate oxidative stress. According to a recent study, chlorogenic acid, quercetin, coenzyme Q10, and silymarin could significantly lower ROS levels by upregulating Nrf2 expression and HO-1 activity [46]. Furthermore, Shi and colleagues suggested that pelargonidin increased the Nrf2 level and decreased ROS production in CCl_4_-induced liver fibrosis [68]. Another study also investigated that adropin protected hepatocytes from oxidative stress by enhancing Nrf2 activity in NASH, further verifying the Nrf2 can reduce oxidative stress and has potential to become dominating effector protein of irisin [69]. As for Nrf2 might play an important role in the antioxidant effect of irisin. Mazur-Bialy et al. discovered that irisin improved the neutralization of ROS in both quiescent macrophages and LPS-stimulated macrophages and that the scavenging of ROS was more effective in LPS-stimulated situations, which confirmed the antioxidant effect of irisin [70]. Furthermore, they demonstrated that irisin performed antioxidant and anti-inflammatory functions by increasing the production of Nrf2 and HO-1 in mouse macrophages, suggesting the Nrf2 is associated with antioxidant effect of irisin [71] (Figure 2). However, the evidence directly reveals that the role of Nrf2 in the antioxidant is still scarce and whether Nrf2 is regulated by irisin and exact molecular mechanism in antioxidant processes need more literature to confirm in the future [71].

### 3.6. Irisin Inhibits JNK Signaling

The c-Jun amino terminal kinase (JNK) signaling pathway is activated by various cellular stresses and participates in the modulation of cell inflammation and oxidative stress [72]. Previous studies showed that the production of ROS was associated with the activation of the JNK signaling pathway. Heo et al. found the upregulation of ER stress and the production of ROS as well as the activation of the JNK signaling pathway in the livers of visfatin-administered mice [30]. Another study demonstrated that hyperuricemia induced the activation of the JNK signaling pathway and caused elevated levels of ROS, indicating that the production of ROS may occur through the JNK signaling pathway [73]. In contrast, Li and colleagues found that guavinoside B alleviated ROS-induced liver damage and reduced the expression of the p-JNK gene, suggesting that negative correlation between JNK expression and oxidative damage [74]. Moreover, it is reported that orientin might activate the JNK signaling pathway to reduce oxidative stress, which offer the direct evidence about the antioxidant effect of JNK signaling pathway [75]. On the other hand, a high level of ROS could induce cancerization though the JNK pathway. Correspondingly, it is found that the overproduction of ROS triggered cholangiocellular carcinogenesis via the JNK signaling pathway [76]. The JNK pathway may participate in the antioxidant effect of irisin. In fact, some investigators observed that irisin inactivated the JNK pathway in vitro [77]. We also found that irisin suppressed oxidative stress by upregulating the activity of telomerase by decreasing the phosphorylation of JNK after hepatic I/R injury [13]. We administered anisomycin, a JNK MAPK inhibitor, to hepatocytes and found that the expression of irisin was not affected but that the activity of telomerase was significantly decreased after hepatic I/R injury, which further confirmed the function of the JNK signaling pathway [13] (Figure 2).

### 3.7. Kindlin-2 Is Involved in Antioxidant Effects of Irisin

Kindlin-2 is a widely expressed and highly conserved integrin-binding protein that is vital for cell-matrix adhesion, cell migration, and signaling [78, 79]. The kindlin family is associated with oxidative stress. Previous researches demonstrated that kindlins could suppress oxidative stress [80]. Furthermore, Guo et al. reported that the depletion of kindlin-2 elevated the production of ROS in human A549 NSCLC cells [81]. Given the role of kindlin-2 in oxidative stress, it could be hypothesized that irisin might control ROS levels by interacting with kindlin-2. Our study showed that irisin improved mitochondrial function, oxidative stress, and ER stress after I/R injury in high-fat diet- (HFD-) fed mice [36]. Notably, kindlin-2 is a main downstream molecule of *α*V*β*5 integrin, and *α*V*β*5 integrin is a receptor of irisin. Considering that irisin combined with *α*V*β*5 integrin alleviates oxidative stress and HIR damage, the possibility that kindlin-2 interacts with irisin to improve oxidative damage is increased [36]. The possibility was fueled when we observed that the inhibition of kindlin-2 by RNAi eradicated the protective effect of irisin on mitochondrial function, ER stress, and oxidative stress [36] (Figure 2). Furthermore, we found that the expression of kindlin-2 was not affected by irisin in HFD-fed mice [36]. In this sense, kindlin-2 collaborated with irisin, and both acted in concert to exert antioxidant effects [36].

### 3.8. GPX4 Participates in the Antioxidant Effect of Irisin

Ferroptosis is a newly discovered cell death that is involved in a variety of disease processes, including NAFLD and ALD [82]. Consequently, the inhibition of ferroptosis is a therapeutic strategy for liver disease. Considering that the excessive accumulation of lipid ROS and increased iron levels are triggers of ferroptosis, antioxidant activity by irisin may be beneficial in liver disease improvement [83]. Glutathione peroxidase 4 (GPX4) is a lipid protective enzyme that eradicates lipid peroxides and prevents the formation of lipid ROS [84]. Furthermore, Yang and colleagues demonstrated that GPX4 was an important upstream modulator of ferroptosis and that GPX4 deficiency enhanced ferroptosis [85]. Overall, the regulation of GPX4 affects ferroptotic activity to ameliorate liver damage. Moreover, we explored the regulatory mechanism of GPX4 by irisin in the livers of septic mice [86]. In our study, we found that GPX4 expression was decreased, but iron levels and ROS levels were elevated in LPS-induced septic mice, indicating ferroptosis occurrence [86] (Figure 2). However, irisin treatment elevated GPX4 expression, markedly decreased ROS production, and decreased iron levels, suggesting that irisin may exert antioxidant effects in a GPX4-dependent manner [86]. It must be noted that when RSL3 was administered to inhibit GPX4 function, the protective effect of irisin disappeared, which further verified that GXP4 was a downstream molecule of irisin and played an essential role in the antioxidant effect of irisin [86]. Intriguingly, Yang et al. found that irisin was a positive regulator of ferroptosis. Irisin decreased GXP4 expression and upregulated ROS production to eliminate cancer cells in pancreatic cancer [87]. The paradoxical results may be a result of disease and tissue differences [87]. Taken together, GXP4 is a target molecule of irisin and participates in oxidative regulation.

### 3.9. SIRT2 Maintains the Stability of Irisin

In the above paragraphs, we demonstrated several antioxidant mechanisms of irisin in liver disease. Obviously, if the serum irisin concentration was low, its antioxidant effect would be impaired in liver disease. Zhang et al. found a negative correlation between serum irisin concentration and triglyceride contents in the liver in obese persons. And the irisin level was also low in NAFLD patients [88], which suggested that low irisin levels were associated with less protective effects. Therefore, the maintenance of normal serum irisin concentrations would also be important for irisin to exert antioxidant effects in liver disease. Furthermore, Li and colleagues found that under lipid stress, the acetylation and ubiquitination of the Fndc5 protein were dramatically strengthened, which accelerated Fndc5 degradation and consequently decreased the serum irisin concentration [89]. Based on this finding, we hypothesized that deacetylation and deubiquitination of Fndc5 would reverse the irisin level reduction. This hypothesis was supported by NAD + -boosting treatment promoting the deacetylation and deubiquitination of Fndc5 and restoring the irisin concentration. Interestingly, the depletion of Sirtuin 2 (SIRT2) diminished the effect of NAD+ and reduced the irisin level, suggesting that SIRT2 was associated with the stability of serum irisin levels. It was speculated that SIRT2 was the deacetylase of Fndc5. Moreover, Li et al. found that the K127/131 and K185/187/189 sites of Fndc5 were essential for the deacetylation activity of SIRT2 [89] (Figure 2). Lantier and colleagues demonstrated that the depletion of SIRT2 would cause abnormal mitochondrial biosynthesis, which might negatively influence the antioxidant effect of irisin [90]. This further confirmed that the interaction between irisin and SIRT2 played an important role in irisin to exert antioxidant effects in liver disease [90].

### 3.10. Other Possible Molecular Targets

There are several different antioxidant mechanisms of irisin that have been discovered in other organs, which implies that irisin might use these mechanisms to alleviate oxidative stress in the liver. In diabetes mellitus, irisin reduces ROS production by inhibiting the PKC-*β*/NADPH oxidase pathway [91]. In cardiovascular diseases, it was reported that irisin elevated the intracellular calcium level and upregulated mitochondrial thermogenesis to preserve cardiac function [92]. Zhang et al. demonstrated that irisin improved the vascular condition by inhibiting ROS/p38 MAPK/NF-*κ*B signaling pathway function [93]. In addition, Wang et al. found that irisin kept the heart from oxidative damage through a SOD2-dependent mitochondrial modulation mechanism. Evidence has shown that irisin protects myocardial cells against I/R-stimulated mitochondrial damage by quelling the mitochondrial permeability transition pore and sustaining the normal mitochondrial membrane potential. In kidney disease, Wu et al. found that irisin can mitigate kidney oxidative injury by activating the FNDC5/irisin-AMPK-Sirt1-PGC-1*α* signaling pathway [94]. In addition, UCP4 can inhibit the production of ROS and sustain the normal level of calcium to avoid apoptosis, which might be a downstream molecule of irisin [95].

## 4. Conclusions

In light of the important role of oxidative stress in liver disease, irisin may be an excellent therapeutic strategy for liver disease treatment. Irisin can decrease ROS production and protect hepatocytes against oxidative stress via several mechanisms. Irisin upregulates autophagy while downregulating ER stress, inflammasome activation, and cell death. Maintaining mitochondrial fusion, fission, and biogenesis balance is also an antioxidant mechanism of irisin. Furthermore, the *α*_v_ integrin, a transmembrane receptor of irisin, combined with irisin to regulate antioxidant signaling pathway. And the molecular basis of the protective effect of irisin is to upregulate UCP2, to attenuate the activation of PRMT3, to elevate the function of telomerase, to activate Nrf2/HO-1 pathway, and to inhibit JNK signaling. Kindlin-2 and GPX4 also participated in the antioxidant effect of irisin. And SIRT2 might maintain the stability of irisin to perform antioxidant function. Moreover, irisin reduced the ROS-induced inflammatory reaction, which is helpful to improve tissue damage. Although the antioxidant effects of irisin have been elucidated and proven by many animal experiments, a lack of clinical data restricts the implementation of this potential therapeutic strategy in clinical practice.

Furthermore, there are few studies clearly demonstrating how irisin regulates cellular oxidative stress in detail, which highlights the importance for more-precise and better-design basic studies to uncover the exact role of irisin in the oxidative stress. On the basis of the current data, it is premature to draw a conclusion that irisin would improve liver diseases outcomes in human body. More well-design prospective clinical studies are imperative to access the usage of irisin as a therapeutic approach in liver diseases, which should be the major research aim for the near future.

## Figures and Tables

**Table 1:** The main mechanism of antioxidant effects of irisin.

Study	Crucial molecules	Liver disease^1^	Animal models	Results
Bi et al. [14], 2019	UCP-2	HIR	C57BL/6J mice^2^	Irisin could suppress the production of ROS via upregulating UCP-2.
Bi et al. [14], 2019	Drp-1, Fis-1	HIR	C57BL/6J mice	Irisin could decrease the expression of Drp-1 and Fis-1 to inhibit inappropriate mitochondrial fission for less oxidative stress.
Bi et al.[14], 2019	PGC-1a, TFAM	HIR	C57BL/6J mice	Irisin might increase the level of the PGC-1a and TFAM to decrease the oxidative stress by promoting mitochondrial biogenesis.
Park et al. [62], 2015	PRMT3	NAFLD	AML12 cells^3^/mouse primary hepatocytes	Irisin may attenuate the function of PRMT3 to decrease the production of ROS.
Fan et al. [27], 2019	NLRP3 inflammasomes	Liver fibrosis	Adult male Sprague-Dawley rats	Irisin may inhibit the formation of the NLRP3 inflammasomes to reduce the hepatic injury due to oxidative stress.
Bi et al. [13], 2020	JNK, telomerase	HIR	Male Sprague-Dawley rats	Irisin would elevate the function of telomerase to promote the autophagy of hepatocyte to reduce the production of ROS via inhibiting JNK phosphorylation.
Zhang et al. [36], 2020	Kindlin-2, *α*_v_ integrins	HIR	C57BL/6J mice	Kindlin-2 may participate in the antioxidant effects of irisin. The *α*_v_ integrins are transmembrane receptor of irisin to exert irisin's antioxidant effect.
Mazur-Bialy and Pocheć [71], 2021	Nrf2, HO-1	NAFLD	Quiescent macrophages/LPS-stimulated macrophages	Irisin reduces oxidative stress via Nrf2/HO-1 involved pathway.
Li et al. [89], 2021	SIRT2	NAFLD	C57BL/6J mice	SIRT2 might maintain the stability of irisin to perform antioxidant function.
Wei et al. [86], 2020	GPX4	Liver in sepsis	C57BL/6J mice	GPX4 is involved in the antioxidant effect of irisin and mitigates the ferroptosis.

^1^The liver disease models established in the experiment or involved in relevant pathways. ^2^C57BL/6J mice is the most used inbred strain of laboratory mouse. ^3^AML12 cells were established from hepatocytes from a transgenic mouse with human TGF-*α*. ROS: reactive oxygen species; NAFLD: nonalcoholic fatty liver disease; HIR: hepatic ischemia reperfusion injury; SOD: superoxide dismutase; UCP-2: Uncoupling protein-2; Drp1: Dynamin-related protein 1; Fis1: fission protein 1; PGC-1a: peroxisome proliferator–activated receptor gamma coactivator-1 alpha; TFAM: mitochondrial transcription factor A; PRMTs: protein arginine methyltransferases; NLRP3: nucleotide-binding oligomerization domain-like receptor 3; TERT: telomerase reverse transcriptase; JNK: c-Jun NH2-terminal kinase; Nrf2: nuclear factor erythroid 2-related factor 2; HO-1: heme oxygenase 1; LPS: lipopolysaccharide; GPX-4: glutathione peroxidase-4; SIRT2: Sirtuin 2.

## Abbreviations

ROS: Reactive oxygen species
RNS: Reactive nitrogen species
NAFLD: Nonalcoholic fatty liver disease
HIR: Hepatic ischemia reperfusion injury
FNDC5: Fibronectin type III domain containing 5
NASH: Nonalcoholic steatohepatitis
SOD: Superoxide dismutase
GSH: Glutathione
UCPs: Uncoupling proteins
UCP-2: Uncoupling protein-2
CIRP: Cold-inducible RNA binding protein
MDA: Malonaldehyde
Drp1: Dynamin-related protein 1
OMM: Outer mitochondrial membrane
Fis1: Fission protein 1
Mff: Mitochondrial fission factor
PGC-1a: Peroxisome proliferator–activated receptor gamma coactivator-1 alpha
TFAM: Mitochondrial transcription factor A
PRMTs: Protein arginine methyltransferases
PA: Palmitic acid
NF*κ*B: Nuclear factor *κ*B
NLRP3: Nucleotide-binding oligomerization domain-like receptor 3
Cpt1a: Carnitine palmitoyltransferase 1a
QDs: Quantum dots
JNK: C-Jun NH2-terminal kinase
MAPK: Mitogen-activated protein kinase
HFD: High-fat diet
ER: Endoplasmic reticulum
Nrf2: Nuclear factor erythroid 2-related factor 2
HO-1: Heme oxygenase 1
LPS: Lipopolysaccharide
iNOS: Inducible nitric oxide synthase
GPX-4: Glutathione peroxidase-4
CAT: Catalase
SIRT2: Sirtuin 2.
